# Supplementary material for: Molecular basis for N-terminal alpha-synuclein acetylation by human NatB
Source: eLife. 2020 Sep 4;9:e57491. doi: 10.7554/eLife.57491 (PMC7494357; doi:10.7554/eLife.57491)
Supplement: Supplementary file 1. — Both the forward and reverse primers for each mutant is indicated. [file elife-57491-supp1.docx]

| **Supplementary File 1. Sequence of primers for preparing mutations** | |
| --- | --- |
| hNAA20- E25A | **Forward:** *GATCCACTTACAGCAACTTATGGGATTC*  **Reverse:** *GAATCCCATAAGTTGCTGTAAGTGGATC* |
| hNAA20- Y27A | **Forward:** *CTTACAGAAACTGCTGGGATTCCTTTC*  **Reverse:** *GAAAGGAATCCCAGCAGTTTCTGTAAG* |
| hNAA20-H73A | **Forward:** *GAATGGCACGGGGCCGTCACAGCTCTG*  **Reverse:** *CAGAGCTGTGACGGCCCCGTGCCATTC* |
| hNAA20-R84A | **Forward:** *GCCCCAGAATTTGCACGCCTTGGTTTGGC*  **Reverse:** *GCCAAACCAAGGCGTGCAAATTCTGGGGC* |
| hNAA20-R85A | **Forward:** *GCCCCAGAATTTCGAGCCCTTGGTTTGGC*  **Reverse:** *GCCAAACCAAGGGCTCGAAATTCTGGGGC* |
| hNAA20-G87A | **Forward:** *TTTCGACGCCTTGCTTTGGCTGCTAAAC*  **Reverse:** *GTTTAGCAGCCAAAGCAAGGCGTCGAAA* |
| hNAA20-N116A | **Forward:** *GTAAGAGTATCTGCCCAAGTTGCAGTT*  **Reverse:** *AACTGCAACTTGGGCAGATACTCTTAC* |
| hNAA20-Y123A | **Forward:** *GCAGTTAACATGGCCAAGCAGTTGGGC*  **Reverse:** *GCCCAACTGCTTGGCCATGTTAACTGC* |
| hNAA20-Y123F | **Forward:** *GCAGTTAACATGTTCAAGCAGTTGGGC*  **Reverse:** *GCCCAACTGCTTGAACATGTTAACTGC* |
| hNAA20-Y137A | **Forward:** *CGGTCATAGAGGCCTATTCGGCCAGCAACG*  **Reverse:** *CGTTGCTGGCCGAATAGGCCTCTATGACCG* |
| hNAA20-Y138A | **Forward:** *CGGTCATAGAGTACGCTTCGGCCAGCAACG*  **Reverse:** *CGTTGCTGGCCGAAGCGTACTCTATGACCG* |
